# Nonoperative Management of a Pediatric Blunt Traumatic Distal Tracheal Injury

**DOI:** 10.1155/crpe/2356477

**Published:** 2026-03-30

**Authors:** Philip Stanic, Emily Vore, Laura Galganski, Charles M. Myer, Erik B. Hysinger, Richard A. Falcone

**Affiliations:** ^1^ College of Medicine, University of Cincinnati, Cincinnati, Ohio, USA, uc.edu; ^2^ Division of Pediatric General and Thoracic Surgery, Cincinnati Children’s Hospital Medical Center, Cincinnati, Ohio, USA, cincinnatichildrens.org; ^3^ Division of Pediatric Otolaryngology-Head and Neck Surgery, Cincinnati Children’s Hospital Medical Center, Cincinnati, Ohio, USA, cincinnatichildrens.org; ^4^ Division of Pulmonary Medicine, Cincinnati Children’s Hospital Medical Center, Cincinnati, Ohio, USA, cincinnatichildrens.org

**Keywords:** blunt tracheal trauma, pediatric tracheal injury, traumatic carina injury

## Abstract

**Introduction:**

Blunt traumatic tracheal injury in children is rare and potentially life‐threatening. Operative management has traditionally been recommended, though recent reports suggest that nonoperative management may be appropriate in selected patients.

**Case Presentation:**

A previously healthy 10‐year‐old boy presented after an unwitnessed helmeted all‐terrain vehicle crash with severe hypoxia, facial swelling, and diffuse subcutaneous emphysema. Initial evaluation at an outside hospital demonstrated bilateral pneumothoraces requiring bilateral chest tube placement. Following transfer, CT imaging revealed a 1 × 1.1 cm posterior tracheal laceration at the carina, pneumomediastinum, pneumopericardium, pneumoperitoneum, extensive subcutaneous emphysema, pulmonary contusions, and rib fractures. Bronchoscopy confirmed a carinal rupture with mediastinal tissue abutting the defect. Attempts to position the endotracheal tube distal to the injury were unsuccessful; therefore, the patient was intubated proximally with a 5.5 Microcuff® tube. Given the patient’s hemodynamic stability, absence of active air extravasation, and ability to maintain ventilation with low pressures, operative repair was deferred. The patient was maintained on low‐pressure ventilation, sedation with paralytics, and empiric antibiotics, with daily bronchoscopy for airway clearance and assessment. The injury progressively granulated, and the patient was successfully extubated 10 days after the injury. He was discharged uneventfully on hospital Day 17 and was clinically asymptomatic at follow‐up.

**Conclusion:**

Severe blunt pediatric tracheal injury may be successfully managed nonoperatively when patients are hemodynamically stable and air extravasation remains controlled and minimal.

## 1. Introduction

Pediatric tracheal injury is an uncommon but potentially life‐threatening injury. Its rarity is partially attributed to protective features of pediatric anatomy, including greater tracheal elasticity and the shielding effect of the mandible over the cervical trachea [1]. When injuries do occur, a large proportion are iatrogenic, secondary to endotracheal intubation or mechanical ventilation, with noniatrogenic cases often resulting from blunt or penetrating trauma [2]. In particular, blunt traumatic tracheal injury is challenging to manage as it is frequently nonisolated, may present only with minimal or nonspecific symptoms such as dyspnea, cough, dysphonia, neck pain, or hemoptysis, and can be overlooked as external findings may not indicate the severity of underlying tracheal injury [2–4]. Additionally, the true incidence of severe traumatic tracheal injury may be underestimated as patients with more extensive traumatic injuries may not survive to reach definitive care.

Historically, early operative intervention has been recommended; however, more recent reports have supported conservative management in selected patients [1, 5–10]. Most reported cases of conservative management involved patients who were managed with mechanical ventilation, with the endotracheal tube (ETT) positioned distal to, or at the level of, the tracheal injury [1, 5, 6, 9, 10]. In contrast, our case involved a distal tracheal injury involving the carina that was successfully managed nonoperatively with proximal, rather than distal, intubation, despite extensive air extravasation at presentation. In addition, serial bronchoscopic examinations documented progressive healing.

This manuscript was prepared following the CARE guidelines (https://www.care-statement.org).

## 2. Case Report

A previously healthy 10‐year‐old male presented to our institution as a transfer from an outside hospital (OSH) after an unwitnessed helmeted all‐terrain vehicle (ATV) crash. At the time of the incident, the patient was responsive but noted to have worsening shortness of breath and significant facial swelling. During the initial emergency medical technician (EMT) assessment, the patient became less responsive and was treated empirically for possible anaphylaxis because of extensive facial swelling. Upon arrival to the OSH emergency department, the patient was hypertensive, tachycardic, unresponsive (Glasgow Coma Scale [GCS] of 3), and cyanotic with significant corporeal and facial edema and diffuse subcutaneous emphysema. He was severely hypoxic (saturations in the 50s) but maintained strong pulses. Due to extensive extremity edema and subcutaneous emphysema, left tibial and left humeral head intraosseous access was established.

With concern for airway compromise, the patient was sedated and intubated with a 6.0 cuffed ETT. Following intubation, the patient remained severely hypoxic. Chest x‐ray was notable for bilateral pneumothoraces with mediastinal shift. Bilateral chest tubes were placed and oxygenation was significantly improved. Following stabilization, computed tomography (CT) of the head was performed and showed no evidence of acute intracranial injury. The patient was then air‐flighted to our facility.

**FIGURE 1:**
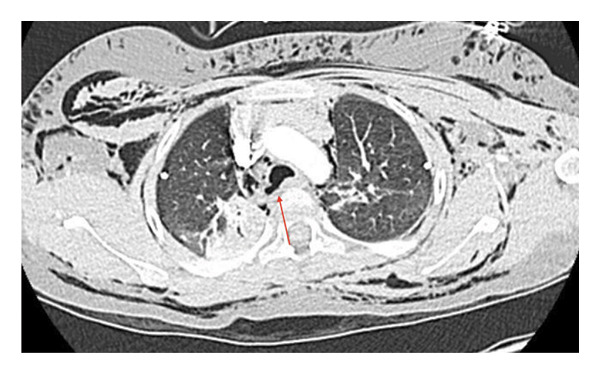
Initial chest computed tomography (CT) performed at our center displaying tracheal defect (arrow).

The patient arrived at our trauma bay hemodynamically stable with saturations in the 90s approximately 2 h after initial OSH presentation. The patient was further evaluated with full body CT revealing a posterior tracheal laceration just above the carina measuring 1 × 1.1 cm, bilateral pulmonary contusions, bilateral traumatic pneumatoceles, pneumomediastinum, pneumopericardium, pneumoperitoneum, and extensive thoracic and abdominal subcutaneous emphysema (Figure 1). Additionally, a mild T1 compression fracture and rib fractures involving the right fourth, left third, and left sixth ribs were identified.

Following multidisciplinary discussion among trauma surgery, otolaryngology, and pulmonology, the decision was made to proceed to the operating room for further airway evaluation with preparedness for emergent surgical intervention. To assess the extent of the tracheal injury, flexible bronchoscopy was performed through the established ETT. Bronchoscopy confirmed a posterior distal tracheal defect involving the carina and communicating with the mediastinum, with a visually estimated luminal opening of at least 0.5 cm (Figure 2(a)). Additionally, mediastinal tissue was visualized abutting the tracheal defect and sealing the injury.

FIGURE 2Bronchoscopic visualization of tracheal defect across hospital course. (a) Hospital day (HD) 1: Central posterior tracheal defect (arrow) that communicates with mediastinum. (b) HD 3: Tissue covering tracheal rupture. (c) HD 5: First visualization of tracheal rupture closure. (d) HD 9: Progressive healing of the trachea rupture. (e) HD 16: Continued healing of the trachea rupture. (f) Site of tracheal rupture 31 days after injury.

**Figure:**
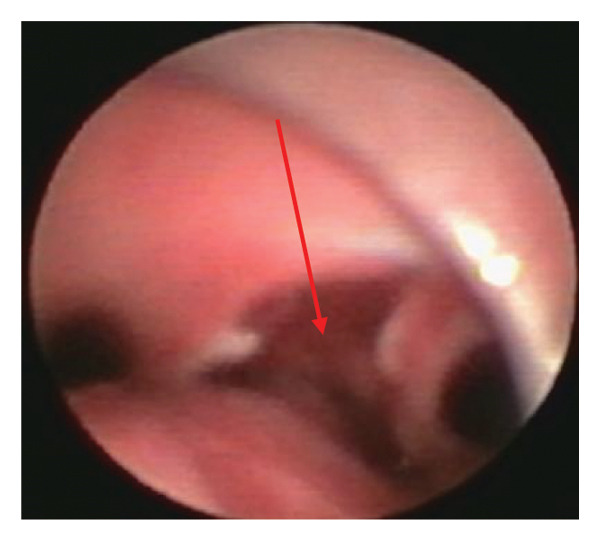
(a)

**Figure:**
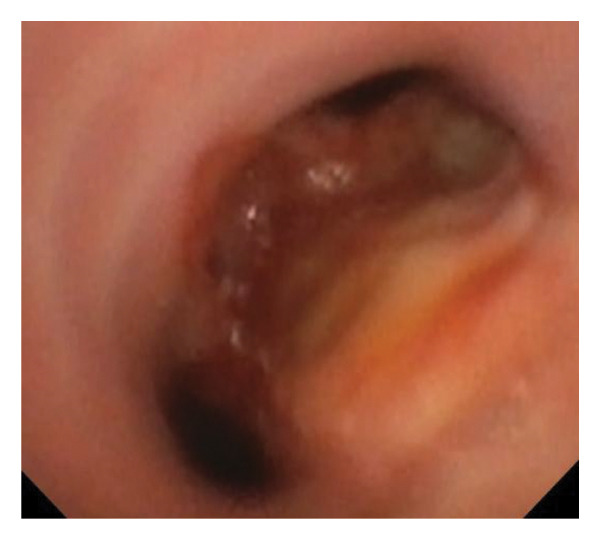
(b)

**Figure:**
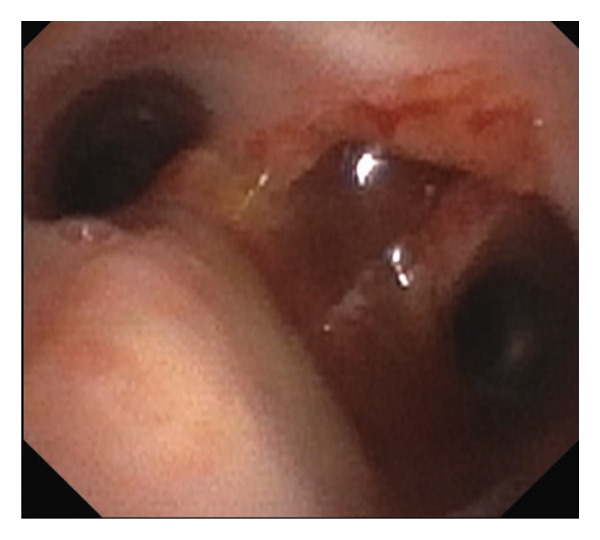
(c)

**Figure:**
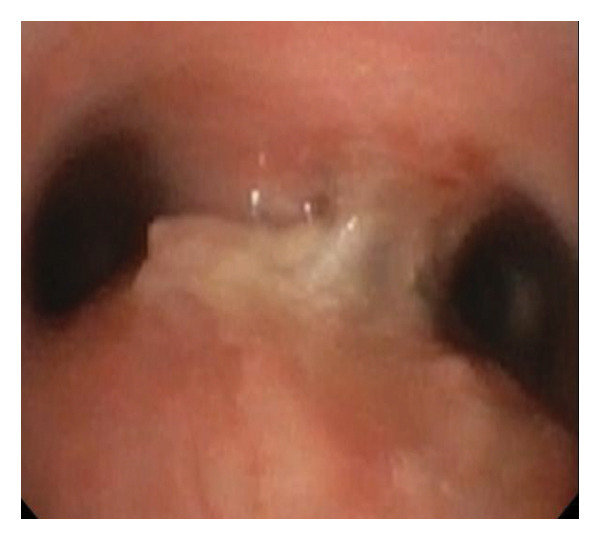
(d)

**Figure:**
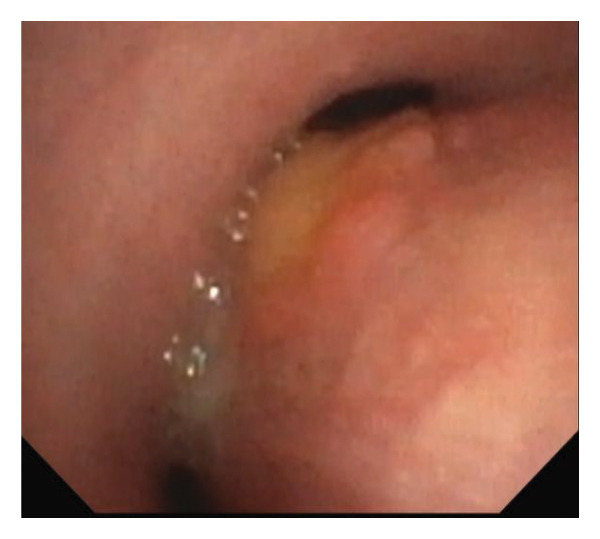
(e)

**Figure:**
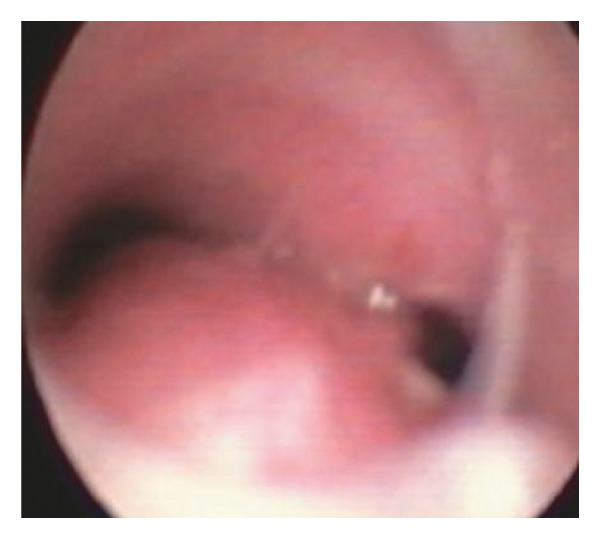
(f)

Intubation of both the left and right mainstem bronchus was attempted to position the ETT distal to the defect but was unsuccessful due to the location of the defect and poor oxygenation, respectively. An attempt was made to inflate the ETT ballon at the site of the injury but appeared to widen the defect. Therefore, the patient was intubated transnasally with placement of a 5.5 Microcuff® ETT proximal to the defect, just below the vocal cords. Due to the location of the tracheal injury, the esophagus was examined with esophagogastroduodenoscopy (EGD) and was found to be uninjured.

Operative vs nonoperative management was discussed among the trauma surgery, otolaryngology and pulmonology teams. It was determined that extracorporeal membrane oxygenation (ECMO) would be required for surgical repair as the lungs could not be ventilated distal to the defect and operative intervention would likely worsen air extravasation. However, given the absence of air leak on chest tube drainage bilaterally, adequate ventilation and oxygenation, and the patient’s hemodynamic stability, surgical management was deferred at that time.

To avoid worsening the tracheal injury and exacerbating air leaks, conventional ventilator settings were limited to a peak inspiratory pressure (PIP) of 18 and positive end‐expiratory pressure (PEEP) of 6. In addition, the patient was kept sedated and paralyzed to minimize physical agitation and to facilitate lower ventilation pressures. Standard airway clearance therapies, such as chest physiotherapy and intrapulmonary percussive ventilation, were avoided, with daily bronchoscopies performed for airway clearance. Empiric broad‐spectrum antibiotic therapy with ceftriaxone and metronidazole was administered for mediastinitis prophylaxis, with no microbial growth identified on respiratory or blood cultures.

Flexible bronchoscopy was initially repeated on hospital day (HD) 2 for airway clearance and tracheal visualization, continuing daily until HD 10. During this time, the patient had intermittent decreases in tidal volumes and hypoxemia due to mucous plugging and endobronchial thrombi that were cleared with bronchoscopy and gentle saline lavage. Daily chest radiographs monitored for pneumothorax stability, ETT location, and chest tube position.

Progressive healing of the tracheal rupture was evident daily with bronchoscopy, with complete sealing of the tracheal defect first noted on HD 5 (Figure 2(c)). With closure of the rupture, vecuronium was discontinued and saline nebulizers were provided on HD 6. Continued tracheal healing and improvements in pulmonary function resulted in successful extubation on HD 10. On HD 11, the patient was weaned to room air and bilateral chest tubes were removed. Surveillance microlaryngoscopy and bronchoscopy (MLB) on HD 16 was notable for the presence of granulation tissue over the tracheal defect and bilateral distal tracheobronchomalacia (Figure 2(e)).

At discharge on HD 17, the patient was alert and interactive, breathing comfortably on room air, tolerating a regular diet, ambulating without difficulty, and had pain well controlled on oral analgesics. The patient was discharged with ciprofloxacin‐dexamethasone nebulizers to decrease granulation tissue and accelerate healing. Follow‐up MLB performed 31 days after injury noted complete healing of the tracheal rupture site, and mild tracheobronchomalacia improved from prior (Figure 2(f)). Six months after discharge, the patient reported an occasional barky cough, likely secondary to residual airway irritation and tracheobronchomalacia, but was otherwise asymptomatic.

## 3. Discussion

Given the rarity of tracheal injuries in the pediatric population, particularly traumatic injuries, management strategies continue to evolve. Using national inpatient data, McCormick et al. identified 106 pediatric laryngotracheal trauma admissions in 2009 alone, of which fewer than 30 were coded as open tracheal injuries, highlighting the limited experience available to guide treatment [11]. Although surgical repair has traditionally been considered the standard approach, successful nonoperative management has been increasingly described in selected patients.

Several algorithms have been proposed for the management of laryngotracheal trauma [12–16]. Initial evaluation should follow advanced trauma life support (ATLS) principles, including primary and secondary surveys, with airway stability guiding management [12, 13, 15, 17]. Tracheostomy may be required at initial assessment for children presenting with acute airway distress, bleeding, or progressive subcutaneous emphysema noted on presentation [12–15]. For those with stable airways, evaluation typically includes flexible fiberoptic endoscopy supplemented by cross‐sectional imaging [12–15]. After endoscopic evaluation, operative intervention, including tracheostomy or surgical exploration, is generally reserved for overt or displaced laryngotracheal fractures, severe mucosal injury, exposed cartilage, or progression of soft‐tissue edema or subcutaneous emphysema [12–16]. In a systematic review, Cheng et al. similarly identified clinical instability, substantial soft‐tissue injury, or fracture of the thyroid, cricoid, or trachea as indications for operative management, although they noted that selected patients with soft‐tissue injury or subcutaneous emphysema may be safely managed without tracheostomy [16].

Overall, across these algorithms, nonoperative management is generally recommended for clinically stable patients without laryngotracheal fractures, or with only nondisplaced fractures, and for those who demonstrate minor soft‐tissue findings with minimal or nonprogressive airway edema or subcutaneous emphysema [12–16]. In a multicenter retrospective study of 20 patients, including many at our institution, Hsu et al. suggest that patients without active air extravasation, who require minimal or no positive‐pressure ventilation, have injury at the site of prior mediastinal surgery, and can be intubated distal to the defect, are appropriate candidates for nonoperative management [17]. Further, in case studies describing successful conservative treatment of tracheal injuries requiring mechanical ventilation, all reported patients were intubated distal to the defect [1, 5, 9, 10]. Positioning the tube distal to the injury is generally thought to reduce mechanical shear at the site of disruption and limit air leak, which may facilitate spontaneous healing. In addition, injuries to the posterior trachea may also respond well to conservative management [16].

Our case is distinct from prior reports in that nonoperative management was successful with the ETT positioned proximal to the tracheal injury. However, despite proximal intubation, nonoperative management likely remained effective because air extravasation was contained by mediastinal tissue and adjunctive measures, such as low peak ventilatory pressures and sedation with paralytics, permitted gradual healing. In addition, appropriate stabilization at the referring hospital and rapid transfer to a center experienced in airway reconstruction contributed to the successful outcome.

Given the severity of the patient’s injuries, daily bronchoscopy was an important component of the nonoperative strategy, allowing serial assessment of airway stability and progressive healing, helping inform the timing of extubation and the possible need for surgical intervention. Bronchoscopy also facilitated airway clearance as conventional airway clearance therapies were avoided to minimize further tracheal injury and air extravasation. However, repeated bronchoscopic evaluation is not without risk and may cause transient airway instability during procedures, airway irritation, and additional mucosal trauma. This strategy is also resource‐intensive and may not be practical in all settings. Therefore, the interval for bronchoscopic reassessment should be tailored to the patient’s clinical condition and the anticipated benefit of serial airway visualization.

## 4. Conclusion

Nonoperative management may be considered in select cases of blunt tracheal injury when the patient is hemodynamically stable and air extravasation is contained.

## Author Contributions

Philip Stanic: writing–original draft and writing–review and editing. Emily Vore Charles M. Myer IV, and Erik B. Hysinger: writing–review and editing. Laura Galganski and Richard A. Falcone Jr.: writing–review and editing and conceptualization.

## Funding

This research did not receive any specific grant from funding agencies in the public, commercial, or not‐for‐profit sectors.

## Disclosure

All authors attest that they meet the current ICMJE criteria for Authorship.

## Consent

Informed consent was obtained from the patient or guardian.

## Conflicts of Interest

The authors declare no conflicts of interest.

## Data Availability

Data sharing is not applicable to this article as no datasets were generated or analyzed during the current study.
